# Post-operative Appendix Specimen Retention Presenting as Small Bowel Obstruction

**DOI:** 10.5811/cpcem.2017.5.34078

**Published:** 2017-10-03

**Authors:** Tiffany Proffitt, Kristen Whitworth, Christopher Trigger

**Affiliations:** Lakeland Health, Department of Emergency Medicine, Saint Joseph, Michigan

## Abstract

One rare complication of appendectomy is a retained appendicolith, which can become a focal point for infection presenting hours to years after surgery. We present a case in which a 50-year-old male presented to the emergency department with a small bowel obstruction one week post appendectomy. A diagnostic laparoscopy was performed, and a necrotic appendiceal specimen containing a staple line across the base as well as an appendicolith was removed. It is crucial to include rare surgical complications in our differentials, alongside the more common pathologies when approaching and treating patients with abdominal pain.

## INTRODUCTION

There is a myriad of post-operative complications from laparoscopic surgery that we as emergency physicians may be prone to dismiss due to the prevalence and less-invasive nature of the procedure. The prevalence of laparoscopic surgery, however, should force us to remain keenly alert and aware of complications in both the acute and chronic state. It is imperative for us to keep the possibility of not only adhesions, but persistent infection of remnants (whether of appendiceal stump or biliary tree), as well as the rarer occurrence of retained surgical specimens, in our differentials for our abdominal pain patients. In some cases these pathologies may not present until decades later.^1^

## CASE REPORT

**Image 1:**
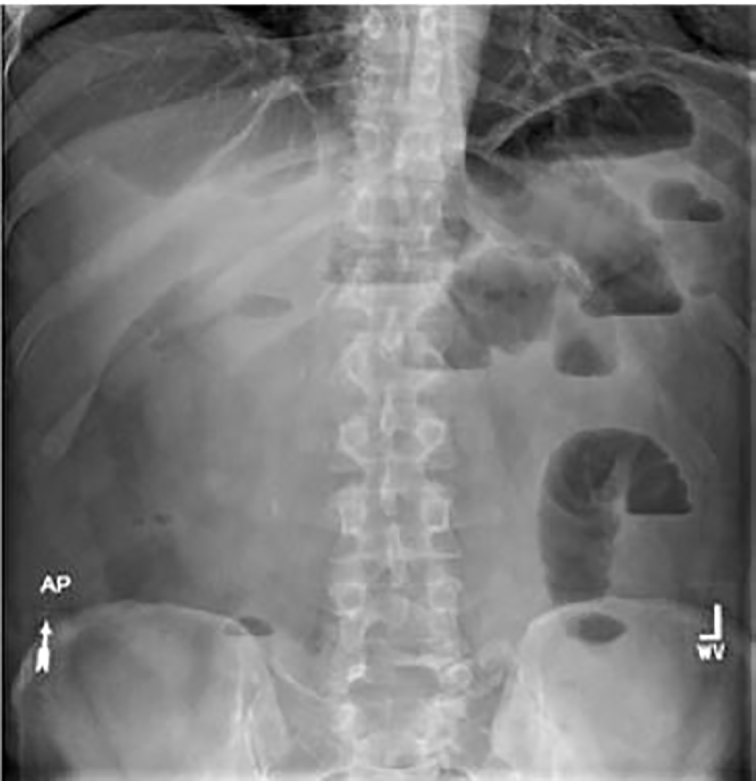
Abdominal radiograph with numerous dilated small bowel loops with air-fluid levels.

A 50-year-old male with a past medical history of ulcerative colitis on azathioprine and mesalamine, *Clostridium difficile* colitis, and hernia repair, presented to the emergency department (ED) with a chief complaint of abdominal pain, nausea and diarrhea. The patient was one-week status post appendectomy performed at an out-of-state hospital. Patient was readmitted to the same hospital hours after the surgery with bloating and abdominal pain, was kept overnight and discharged the next day. Since the surgery, the patient had been experiencing worsening abdominal pain, distention, and nausea, with one bout of emesis a few days prior to presentation in our ED. On the day of presentation, the patient experienced three episodes of water diarrhea, night sweats without fever, and intermittent passage of flatus. An outpatient acute abdominal series performed the morning of presentation demonstrated numerous dilated small bowel loops with air-fluid levels suggestive of small bowel obstruction with no free air (Image 1).

**Image 2:**
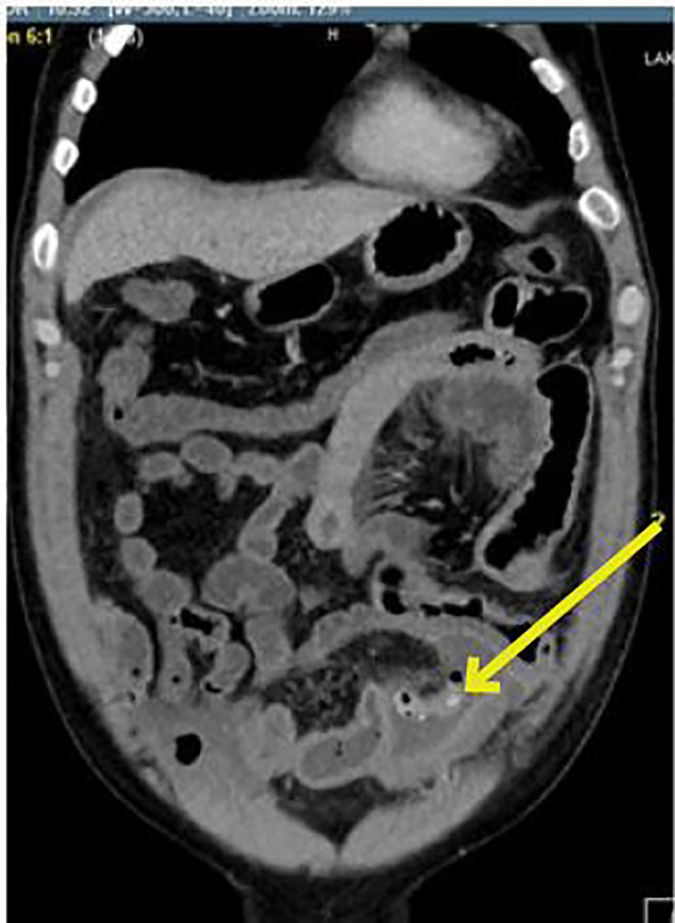
Coronal computerized tomography of the abdomen and pelvis. An air-filled tubular density with a single calcification and staple line is visible adjacent to a distended loop of small bowel (arrow).

Physical exam revealed an afebrile, uncomfortable-appearing male with abdominal distension and decreased bowel sounds throughout. He had generalized tenderness to palpation with no guarding or rigidity. Despite this presentation, his vital signs and initial blood work were unremarkable. A one-liter normal saline fluid bolus was administered and a nasogastric tube was placed with 200mL of nonbilious output. The patient refused anti-nausea or pain medication. After discussion with the general surgeon, computerized tomography (CT) of the abdomen and pelvis with intravenous (IV) contrast was performed for further investigation of a suspected intra-abdominal abscess in addition to a small bowel obstruction. Imaging demonstrated abnormal wall thickening along the cecum with dense fluid within the cecum concerning for hemorrhage, as well as a mildly distended small bowel loop in the left anterior abdomen. There was a surgical staple line adjacent to the inferior portion of the loop. Contiguous with the staple line was a small tubular density filled with air and a single calcification (Image 2).

**Image 3:**
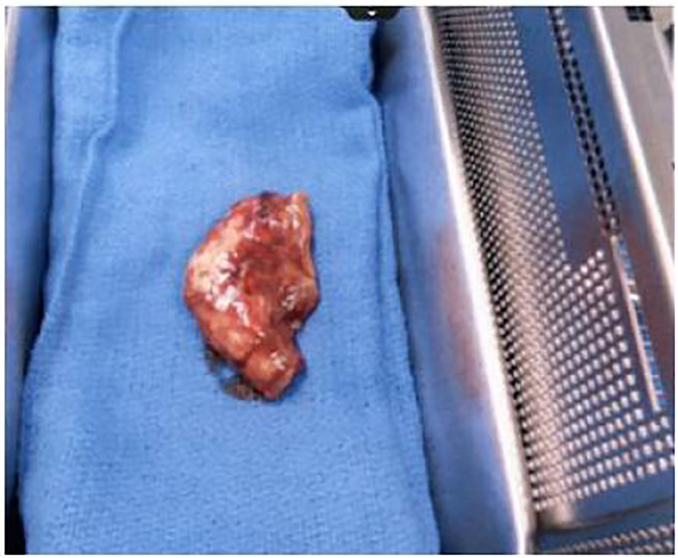
The specimen retrieved in the operating room was a necrotic appendix.

Based upon these findings, there was concern for retained appendix in addition to small bowel obstruction and intra-abdominal abscess. Antibiotic coverage with IV piperacillin/tazobactam was started and the patient was admitted to general surgery. Diagnostic laparoscopy was performed that day, and in the operating room inflammation of the parietal peritoneum was noted as was a large purulent pocket located below the patient’s largest prior periumbilical port site. Additionally, a necrotic appendix was visualized and removed. Further examination demonstrated that the appendix specimen contained a staple line across the base of the appendix containing an appendicolith (Image 3). The patient was discharged seven days post operatively after resolution of a post-operative ileus. He followed up outpatient with general surgery eight days after discharge and reported no further complaints. Pathology report from the specimen retrieved during surgery was consistent with appendiceal tissue.

## DISCUSSION

Appendicitis is a common surgical emergency with over 270,000 appendectomies performed in the United States each year.^2^ Although open appendectomies are still performed, the laparoscopic approach is becoming the preferred treatment of acute appendicitis. Benefits of laparoscopic appendectomy include shorter hospital stays, decreased requirement of postoperative analgesia, and earlier return to work when compared with open appendectomy.^3^ While laparoscopic appendectomies are less likely to have wound infections, the rate of intra-abdominal abscesses is increased compared to open appendectomies.^4^

**CPC-EM Capsule**

What do we already know about this clinical entity?

Appendicitis is a common surgical emergency with over 270,000 appendectomies performed in the U.S, each year. Two rare complications of appendectomy are stump appendicitis and retained appendicoliths.

What makes this presentation of disease reportable?

Over the past 40 years, there have been 30 reported cases in the literature of retained appendicoliths causing intra-abdominal abscesses.

What is the major learning point?

Retained appendicoliths can have unusual presentations and present with complications days to years after surgery. We present a rare presentation of small bowel obstruction caused by an intra-abdominal abscess that developed from a retained appendicolith.

How might this improve emergency medicine practice?

This case serves as an important reminder to include these rare surgical complications in our differentials, alongside the more common pathologies, when approaching and treating patients with abdominal pain.

Two rare complications of appendectomy are stump appendicitis and retained appendicoliths. Patients may present hours to years after surgery. Stump appendicitis is caused by a retained appendicular stump. A 60-year review of medical literature found a total of 57 reported cases of stump appendicitis.^5^ Presentation is similar to appendicitis, although it is not usually considered in differential diagnoses as patients report a history of a prior appendectomy. Of the known cases, patients presented a mean of 108 months after appendectomy; 34.5% of cases had been performed with a laparoscopic approach and 65.5% had undergone an open appendectomy.^5^ Treatment requires surgery to remove the remnant of the appendiceal base.

An appendicolith is a collection of fecal debris and calcium salts residing in the appendix that can lead to acute appendicitis. With increased use of CT, they are becoming a more common incidental finding. An extraluminal fecalith, however, is a focal point for infection and should cause concern for appendiceal perforation. Over the past 40 years, there have been 30 reported cases in the literature of retained fecaliths causing intra-abdominal abscesses.^6^ Appendicoliths can be retained secondary to rupture of the appendix prior to surgery or from failure of their removal during surgery. Retained appendicoliths can have unusual presentations with one documented case of an empyema caused by an appendicolith that ventured into the chest cavity.^7^ Other unusual cases include a tubo-ovarian abscess secondary to migration of the appendicolith into the right fallopian tube and a case presenting as a retroperitoneal abscess two-years status post laparoscopic appendectomy.^8^,^1^ Management of retained fecaliths requires drainage of the abscess and surgical removal. More recently, there have been case reports of management with CT-guided drainage and percutaneous removal of the appendicolith using a basket retrieval device.^9^

## CONCLUSION

As emergency physicians, it is crucial for us to include these rare surgical complications in our differentials, alongside the more common pathologies when approaching and treating our patients with abdominal pain.
